# Safety, pharmacokinetics and pharmacodynamics of a novel γ-aminobutyric acid (GABA) receptor potentiator, HSK3486, in Chinese patients with hepatic impairment

**DOI:** 10.1080/07853890.2022.2129433

**Published:** 2022-10-10

**Authors:** Yue Hu, Xiaojiao Li, Jingrui Liu, Hong Chen, Wenbo Zheng, Hong Zhang, Min Wu, Cuiyun Li, Xiaoxue Zhu, Jinfeng Lou, Pangke Yan, Nan Wu, Xiao Liu, Shiping Ma, Xu Wang, Yanhua Ding, Chengluan Xuan

**Affiliations:** a Phase I Clinical Trial Unit, First Hospital, Jilin University, Jilin, China; b Haisco Pharmaceutical Group, Sichuan, China; c Department of Anesthesiology, First Hospital, Jilin University, Jilin, China

**Keywords:** Efficacy, hepatic impairment, HSK3486, pharmacokinetics, safety

## Abstract

**Background:**

The primary objective of this study was to investigate if hepatic impairment alters the safety, pharma**c**okinetics, and pharmacodynamics of HSK3486.

**Research design and methods:**

This was a clinical trial of HSK3486 in subjects with normal hepatic function (*n* = 8), and mild (Child-Pugh A; *n* = 8), or moderate (Child-Pugh B; *n* = 8) hepatic impairment. Each subject received an IV bolus dose of 0.4 mg/kg HSK3486 for 1 min, immediately followed by a maintenance infusion of 0.4 mg/kg/h HSK3486 for 30 min.

**Results:**

In total, 24 subjects were enrolled and completed the study. HSK3486 was generally well tolerated by all subjects. There were no serious AEs and no deaths reported during the study. The incidence of AEs was numerically highest in subjects with moderate hepatic impairment. The exposure (AUC) of HSK3486 increased gradually with the decrease in hepatic function; however, degree of hepatic impairment had little effect on HSK3486 PD (MOAA/S and BIS).

**Conclusions:**

Overall, there were no clinically relevant differences in HSK3486 exposure or PD in subjects with mild or moderate hepatic impairment compared to normal control. These data imply that HSK3486 dose adjustment is not warranted in subjects with mild or moderate hepatic impairment.

**Trial registration:**

The trial is registered at ClinicalTrials.gov (CT.gov identifier: NCT04145596).

**Key Message**
- HSK3486 at an IV bolus dose of 0.4 mg/kg and a maintenance infusion of 0.4 mg/kg/h was safe and well tolerated by all mild or moderate hepatic impairment subjects and normal hepatic function subjects.
- There were no clinically relevant differences in HSK3486 exposure or PD in subjects with mild or moderate hepatic impairment compared to subjects with normal hepatic function.
- HSK3486 dose adjustment is not required in subjects with mild or moderate hepatic impairment.

## 1. Introduction

**Figure 1.**
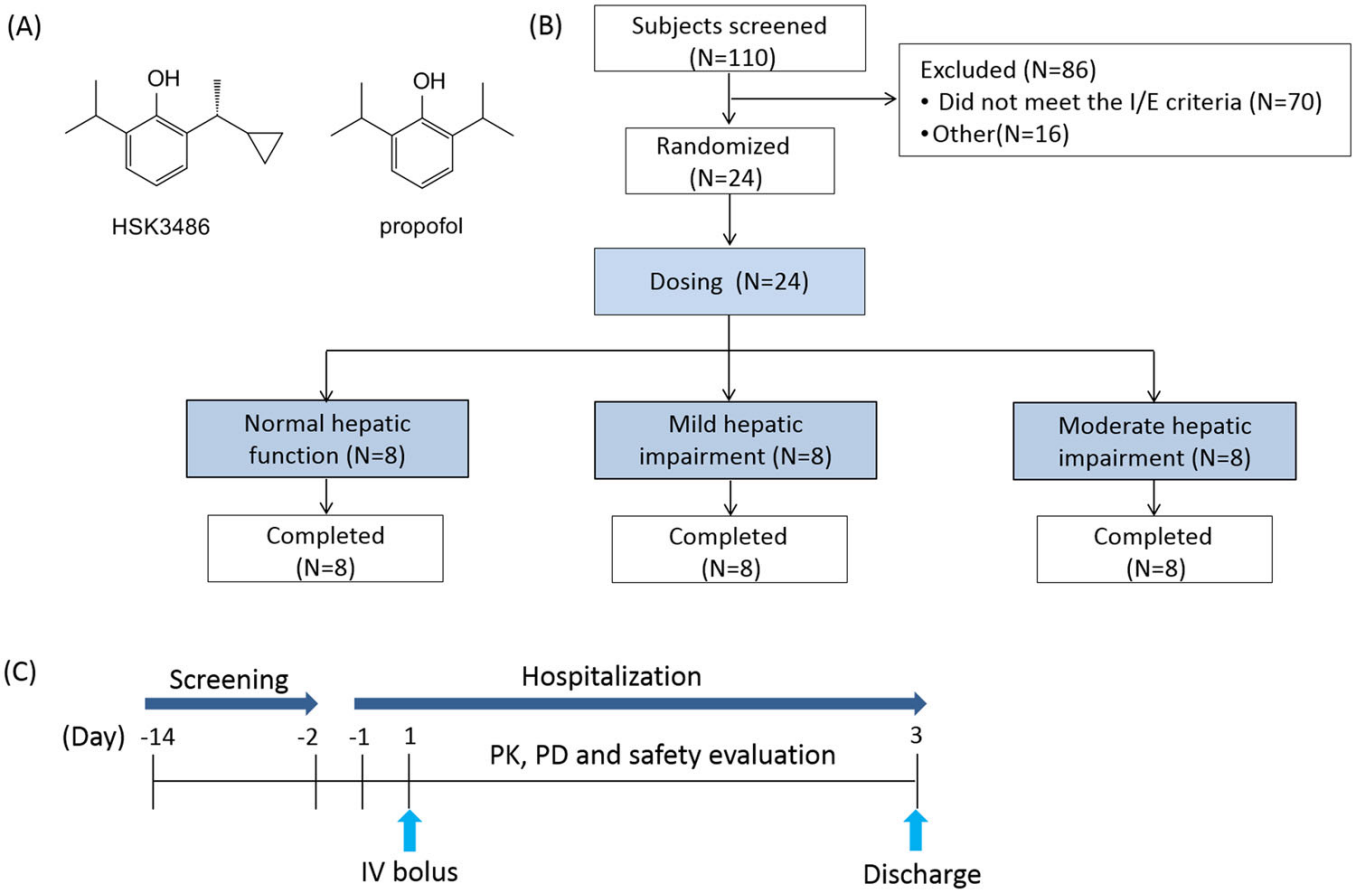
Chemical structure of HSK3486 and propofol (A), study design and flow chart (B and C).

HSK3486 is a novel 2,6-disubstituted phenol derivative and a γ-aminobutyric acid (GABA) receptor potentiator that is similar to propofol. HSK3486 is a candidate intravenous drug that was developed for anaesthesia induction and maintenance (Figure 1) [1–3]. HSK3486 has been investigated in several Phase I to III clinical trials that enrolled approximately 500 subjects, including healthy subjects, intensive care unit patients and those undergoing fibreoptic bronchoscopy, colonoscopy, gastroscopy, or elective surgery [2, 4–6]. Results showed that the clinical characteristics of HSK3486 were comparable to propofol; the potency of HSK3486 was equivalent to propofol at 1/4 − 1/5 of the dosage; the plasma-level time curve for HSK3486 resembled that of propofol, and may be divided into three phases, an initial distribution phase (t_1/2α_=2.0 min), a subsequent redistribution phase (t_1/2β_=34.9 min) and a terminal elimination phase (t_1/2γ_=6.2 h) [2]. HSK3486 exhibited non-inferiority anaesthesia/sedation compared to propofol in patients undergoing fibreoptic bronchoscopy and has clinical advantages compared to propofol, including a significant reduction in injection pain and lower hypotension rate [4–11].

Similar to propofol, HSK3486 is extensively metabolised in the liver by UDP-glucuronosyltransferases (UGTs) and CYP enzymes to produce inactive metabolites. The major circulating metabolite of HSK3486, M4 (79.3%), is a nonhypnotic and non-toxic glucuronidation product that is excreted through urine. Previous *in vitro* studies imply CYP2B6 is the major CYP enzyme that mediates HSK3486 metabolism [2, 12].

The liver is important for drug elimination. Hepatic impairment alters hepatic blood flow, plasma protein binding and biliary excretion, and modifies drug metabolism, reduces drug clearance, and influences drug pharmacokinetics (PK) [13–17]. Hence, the PK of HSK3486 may be altered in patients with hepatic impairment. As the patient population of HSK3486 could include people with mild or moderate hepatic impairment, it is important to determine the impact of hepatic impairment on the metabolism of HSK3486.

The objective of the study was to determine the safety, plasma PK, and pharmacodynamics (PD) of HSK3486 in participants with varying hepatic impairment compared with normal controls.

## 2. Materials and methods

### 2.1. Study management and registration

This was a single-site clinical trial conducted from November 07, 2019 (the first subject screening visit) to April 20, 2020 (the last subject last visit). The protocol was approved by the Jilin University First Affiliated Hospital Ethics Committee. The clinical trial (registration No.: NCT04145596, https://clinicaltrials.gov/) was conducted in accordance with ICH GCP guidelines and the World Medical Congress Declaration of Helsinki. Written informed consent was provided by all study subjects.

### 2.2. Subjects

Men and women (18–64 yr) with a body mass index (BMI) between 18 and 30 kg/m^2^; normal liver function group subjects have no previous primary diseases with normal or non-clinically laboratory abnormalities; liver insufficiency of Child Pugh grade A or B patients results from primary liver disease, including viral hepatitis type B and C, non-alcoholic steatohepatitis; modified Mallampati score was I or II and Allen test negative were eligible for this study. Subjects were excluded if they had a had a history of alcohol or drug abuse, or were smokers. history of chronic smoking or drug and/or alcohol abuse; history of liver failure, or cirrhosis complicated with hepatic encephalopathy, hepatocellular carcinoma, or rupture and bleeding of oesophageal and/or gastric varices. Mean body weight and age of subjects with normal hepatic function must have been within ±10 kg and ±5 yrs, respectively, of the mean body weight and age of subjects with mild and moderate hepatic impairment.

### 2.3. Study execution

Participants with mild hepatic impairment (Child-Pugh A; *n* = 8), moderate hepatic impairment (Child-Pugh B; *n* = 8) and matched control participants with normal hepatic function (*n* = 8) were enrolled. All subjects were given an IV bolus dose of 0.4 mg/kg HSK3486 for 1 min, immediately followed by 0.4 mg/kg/h maintenance for 30 min (Figure 1).

Subjects were admitted to the study site on Day-1 and received study drug on Day 1 after a minimum 8 h overnight fast. Water was withdrawn 2 h prior to dosing. Participants were dosed in a dedicated treatment room equipped with monitoring and emergency equipment. Additional oxygen (2-10 L/min) was delivered *via* an oxygen mask during study drug administration. During treatment, blood pressure measurements, pulse oximetry, and 3-lead electrocardiogram were used to measure vital signs, and bispectral index (BIS) (BIS VISTA™ monitor; Aspect Medical Systems, Norwood, MA, USA) was monitored. After safety assessments, participants were discharged on Day 3.

### 2.4. Safety

The Common Terminology Criteria for Adverse Events (CTCAE) v5.0 were used to classify the severity of adverse events (AEs). Vital sign measurements, 3-lead and 12-lead ECG, clinical laboratory tests and physical examination were also as the part of the safety assessment. Pain intensity at the injection site was evaluated. AEs were categorized by severity and relationship to study drug. AEs of special interest were sedative-related AEs, including hypoxia, hypotension, bradycardia, and apnoea.

### 2.5. Pk analysis

Arterial blood samples for PK analysis (4 mL each) were collected at pre-dose, at the end of the loading dose infusion (1 min after the start of infusion), 5, 10, 20 and 30 min after the start of maintenance dose infusion, and 1, 2, 4, 8, 15, 30 min and 1 h after the termination of infusion. Venous blood samples were collected 2, 3, 4, 6, 8, 12, 24, and 48 h after the termination of infusion. Additional samples (5 mL) for evaluating plasma protein binding were collected 1 min after the start of infusion and 1 min after the termination of infusion. Blood samples were drawn into blood collection tubes containing K_2_EDTA. The samples were centrifuged and stored at −80**°**C until analysis . Urine samples were collected within 48 h after drug administration, and maintained at −80**°**C until analysis.

Concentrations of HSK3486 in plasma and M4 in plasma and urine were quantified by a fully validated liquid chromatography-tandem mass spectrometry (LC-MS/MS) method. The calibration range was 5.0 ∼ 5000 ng/mL for HSK3486 and 0.8 ∼ 2000 ng/mL for plasma protein binding of HSK3486. Accuracy was −0.7%∼7.3% for HSK3486 and −1.9%∼ 4.4% for plasma protein binding of HSK3486. Precision was within 6.6% CV for HSK3486 and 3.6% CV for plasma protein binding of HSK3486. The calibration range was 5.0 ∼ 5000 ng/mL for plasma M4 and 10.0 ∼ 10000 ng/mL for urine M4. Accuracy was −2.0%∼4.0% for plasma M4 and −1.8%∼ 4.4% for urine M4. Precision was within 5.9% CV for plasma M4 and 5.2% CV for urine M4.

### 2.6. PD analysis

BIS monitoring and MOAA/S scores were used to evaluate the clinical effect of HSK3486. BIS VISTA™ monitor was used to monitored the BIS scores, which were recorded at 0 h and thereafter once every minute until 60 min post-dose. MOAA/S scores were recorded at 0 h, after the termination of the loading dose infusion, every 5 min during the maintenance dose infusion, and then every 2 min until full recovery, defined as three consecutive MOAA/S scores of 5.

### 2.7. Statistics

Sample size was determined based on National Medical Products Administration (NMPA) guidance for the conduct of PK studies. A total of 8–12 subjects were considered sufficient to evaluate the PK characteristics of the study drug; therefore, eight subjects were allocated to each cohort.

Statistical analyses were performed using SAS 9.4 and PK parameters were calculated by a noncompartmental method using WinNonLin®, version 8.3.1 (Certara, Princeton, NJ, USA). The impact of hepatic impairment versus the control group was investigated using analysis of variance (ANOVA), which included hepatic function as a fixed effect, and the corresponding 90% confidence intervals (CIs) around the geometric least-squares mean (GLSM) ratio of C_max_, AUC_0-t_ and AUC_0-inf_ were calculated. HSK3486 exposure with respect to time until fully alert and BIS parameters were represented as scatter plots.

## 3. Results

### 3.1. Subjects

Among 110 subjects that were screened, 24 subjects (normal hepatic function, *n* = 8; mild hepatic impairment (Child-Pugh A), *n* = 8; moderate hepatic impairment (Child-Pugh B), *n* = 8) were enrolled and completed the study. Modified Mallampati score was I or II and Allen’s test were all negative. Subjects’ mean height, weight, BMI, age and gender ratio were similar across the three cohorts (Table 1).

**Table 1.** Subjects’ demographic and baseline clinical characteristics.

	Normal hepatic function	Mild hepatic impairment	Moderate hepatic impairment	Total
	(*N* = 8)	(*N* = 8)	(*N* = 8)	(*N* = 24)
Age (yr)				
Mean (SD)	49.3 (3.41)	46.6 (6.91)	55.5 (4.50)	50.5 (6.22)
Median (Min, Max)	49 (46, 55)	45 (35, 57)	55.5 (50, 64)	50 (35, 64)
Sex, *n* (%)				
Male	6 (75.0)	7 (87.5)	6 (75.0)	19 (79.2)
Female	2 (25.0)	1 (12.5)	2 (25.0)	5 (20.8)
Height, cm, mean (SD)	165.25 (6.337)	168.26 (11.272)	163.61 (10.251)	165.71 (9.314)
Weight, kg, mean (SD)	66.23 (4.960)	68.80 (7.979)	65.66 (9.938)	66.90 (7.673)
BMI, kg/m^2^, mean (SD)	24.6 (1.77)	24.9 (2.10)	24.9 (1.73)	24.8 (1.79)
Albumin, g/L, mean (SD)	39.71 (2.564)	42.30 (2.364)	33.61 (6.421)	38.54 (5.486)
Airway evaluation, *n* (%)				
I	2 (25.0)	3(37.5)	5 (62.5)	10 (41.7)
II	6 (75.0)	5(62.5)	3 (37.5)	14 (58.3)
Allen’s test, *n* (%)				
Negative	8 (100)	8 (100)	8 (100)	24 (100)
Positive	0	0	0	0
Child–Pugh score, *n* (%)				
5–6 (mild)	0	8 (100)	0	8 (33.3)
7–9 (moderate)	0	0	8 (100)	8 (33.3)
10–15 (severe)	0	0	0	0
Encephalopathy grade, *n* (%)				
None	8 (100)	8 (100)	8 (100)	24 (100)
1–2	0	0	0	0
3–4	0	0	0	0
Ascites, *n* (%)				
Absent	8 (100)	8 (100)	2 (25)	18 (75)
Slight	0	0	4 (50)	4 (16.7)
Moderate	0	0	2 (25)	2 (8.3)
Bilirubin (μmol/L), *n* (%)				
	8 (100)	8 (100)	4 (50)	20 (83.3)
<34.2, 34.2-51.3, >51.3	0	0	2 (25)	2 (8.3)
	0	0	2 (25)	2 (8.3)
Albumin (g/L), *n* (%)				
≥35	8 (100)	8 (100)	3 (37.5)	19 (79.2)
≥35, 28-34, <28	0	0	3 (37.5)	3 (12.5)
<28	0	0	2 (25)	2 (8.3)
Prolonged prothrombin time (s), *n* (%)				
	8 (100)	8 (100)	8 (100)	24 (100)
<4, 4-6, >6	0	0	0	0
	0	0	0	0
INR, mean (SD)	0.920 (0.0378)	1.046 (0.0831)	1.124 (0.1423)	1.030 (0.1267)

BMI: body mass index; INR: international normalised ratio.

### 3.2. Safety and tolerability

Seventeen subjects (70.8%) experienced a total of 35 AEs, with 30 AEs among 17 subjects possibly related to the study drug. A total of 5 participants (62.5%), 7 participants (87.5%) and 5 participants (62.5%) with mild, moderate hepatic impairment and normal control group experienced drug-related AEs, respectively. The most common drug-related AEs were hypotension, experienced by 25% of subjects with all three treatment groups; respiratory depression, experienced by 12.5% of subjects with normal hepatic function, 25% of subjects with mild hepatic impairment, and 50% of subjects with moderate hepatic impairment; platelet count decreased, experienced by 0% of subjects with normal hepatic function, 12.5% of subjects with mild hepatic impairment, and 25% of subjects with moderate hepatic impairment; and anaemia, experienced by 0% of subjects with normal hepatic function or mild hepatic impairment, and 25% of subjects with moderate hepatic impairment (Table 2). Sedative-related AEs were experienced by 37.5% of subjects with normal hepatic function and 25% of subjects with mild hepatic impairment or moderate hepatic impairment.

**Table 2.** Summary of adverse events.

	Normal hepatic function (*N* = 8)	Mild hepatic impairment (*N* = 8)	Moderate hepatic impairment (*N* = 8)	Total (*N* = 24)
	*n* (%)	*n* (%)	*n* (%)	*n* (%)
All AEs	5 (62.5)	5 (62.5)	7 (87.5)	17 (70.8)
Drug-related AEs	5 (62.5)	5 (62.5)	7 (87.5)	17 (70.8)
All SAEs	0	0	0	0
Sedative-related AEs	3 (37.5)	2 (25.0)	2 (25.0)	7 (29.2)
Respiratory depression	1 (12.5)	2 (25.0)	4 (50.0)	7 (29.2)
Anoxia	1 (12.5)	0	0	1 (4.2)
Platelet count decreased	0	2 (25.0)	2 (25.0)	4 (16.7)
Total bile acid increased	0	0	1 (12.5)	1 (4.2)
Neutrophil count decreased	0	0	1 (12.5)	1 (4.2)
Blood bilirubin elevated	0	0	1 (12.5)	1 (4.2)
Diastolic hypotension	1 (12.5)	0	0	1 (4.2)
Urine glucose positive	0	1 (12.5)	0	1 (4.2)
Lymphocyte count decreased	0	0	1 (12.5)	1 (4.2)
Amylase elevated	0	1 (12.5)	0	1 (4.2)
White blood cell count decreased	0	0	1 (12.5)	1 (4.2)
Hypotension	2 (25.0)	2 (25.0)	2 (25.0)	6 (25.0)
Lipase elevated	0	0	1 (12.5)	1 (4.2)
Hyperglycaemia	0	0	1 (12.5)	1 (4.2)
Hypoalbuminemia	0	0	1 (12.5)	1 (4.2)
Anaemia	0	0	2 (25.0)	2 (8.3)
Anaesthetics airway complications	1 (12.5)	0	0	1 (4.2)
Haematuria	0	0	1 (12.5)	1 (4.2)

*n*: the subject number; %: the percentage of the AE.

All AEs were mild to moderate in severity, except one Grade 3 AE, platelet count decreased (drug-related), which was experienced by a subject with moderate hepatic impairment. All Grade 2 or 3 AEs were experienced by subjects with moderate hepatic impairment who had underlying liver disease or abnormal laboratory test results at baseline. There were no deaths, serious AEs, AEs led to withdrwal during the study. No subject reported notable pain at the injection site.

**Figure 2.**
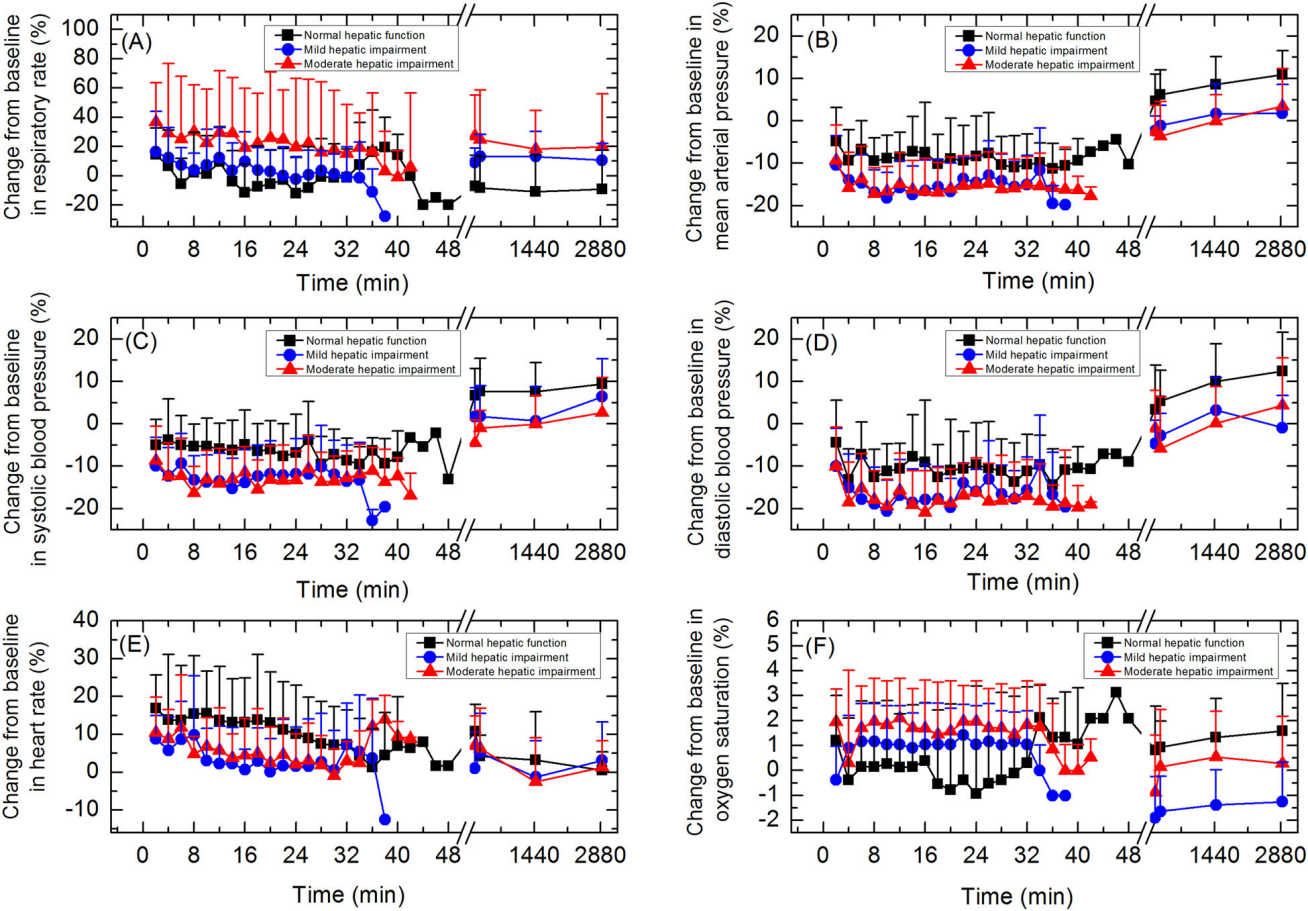
Vital signs: (A) respiration rate; (B) mean arterial pressure; (C) systolic blood pressure; (D) diastolic blood pressure; (E) heart rate; (F) oxygen saturation (SpO2). Data are presented as mean＋standard deviation.

Change from baseline in vital signs during treatment and follow up were similar across subjects with normal hepatic function, mild hepatic impairment or moderate hepatic impairment (Figure 2).

AEs related to vital signs were experienced by 62.5% of subjects. AEs related to vital signs included respiratory depression, experienced by 29.2% of subjects, hypotension, experienced by 20.8% of subjects, anoxia, experienced by 4.2% of subjects, and diastolic hypotension, experienced by 4.2% of subjects. Severity of all AEs related to vital signs was Grade 1, except for one Grade 2 AE, hypotension, experienced by a subject with moderate hepatic impairment. This subject was administered drug therapy (norepinephrine) and non-drug therapy (fluid infusion). One subject who experienced respiratory depression and anaesthetic airway complications recovered after non-drug therapy (jaw lifting and sputum aspiration). Other subjects who experienced AEs related to vital signs recovered spontaneously without treatment

### 3.3. Pharmacokinetic properties

**Figure 3.**
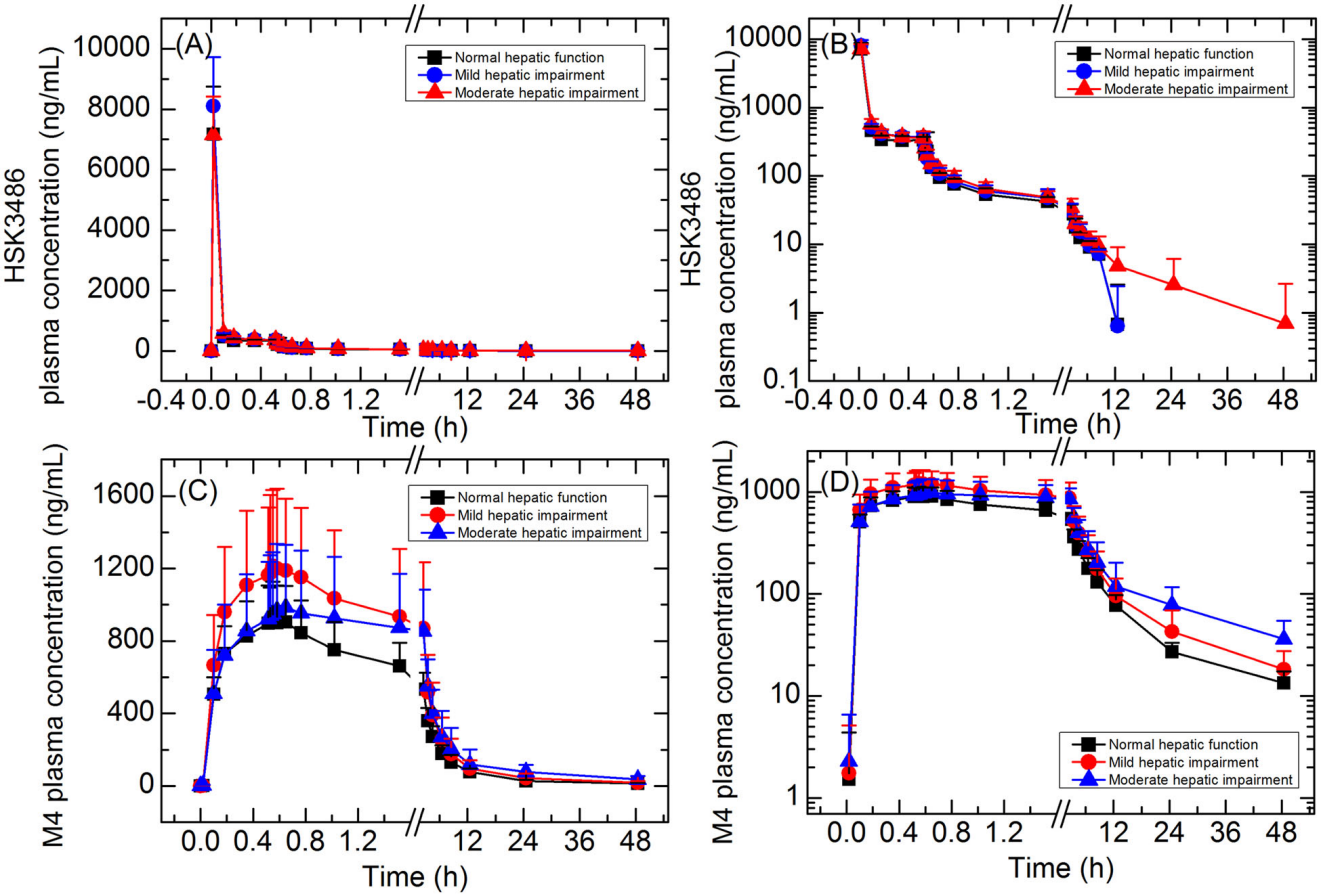
Plasma concentration-time curves for HSK3486 and its metabolite M4 in linear (A, C) and semi-log scale (B, D). Data are presented as mean＋standard deviation.

Mean HSK3486 and the metabolite M4 plasma concentrations over time for subjects with mild and moderate hepatic impairment and matched normal hepatic function, are shown in Figure 3, and total PK parameters are summarised in Table 3.

**Table 3.** Pharmacokinetic characteristics of total and unbound HSK3486 and the metabolite M4.

Analyte	Parameters	Normal hepatic function (*N* = 8)	Mild hepatic impairment (*N* = 8)	Moderate hepatic impairment (*N* = 8)
Total HSK3486	C_max_ (ng/mL)	7170.0 (22.07)	8105.0 (19.92)	7141.3 (17.87)
	T_max_ (h)	0.02 (0.02, 0.02)	0.02 (0.02, 0.02)	0.02 (0.02, 0.02)
	t_1/2_ (h)	4.17 (37.56)	3.57 (34.59)	13.2 (102.44)
	AUC_0-t_ (ng*h/mL)	590.6 (13.97)	663.9 (16.57)	736.9 (27.21)
	AUC_0-inf_ (ng*h/mL)	631.6 (13.98)	698.3 (16.26)	849.0 (34.75)
	CL (L/h)	64.0 (15.46)	59.9 (13.8)	50.9 (35.72)
	wn_CL ((L/h)/kg)	0.97 (14.77)	0.88 (16.22)	0.77 (28.68)
	V_d_ (L)	375.3 (32.88)	305.5 (34.54)	755.3 (73.57)
	wn_V_d_ (L/kg)	5.69 (33.25)	4.47 (31.86)	11.5 (75.42)
	V_dss_ (L)	105.3 (29.75)	80.1 (28.26)	308.4 (97.15)
	wn_V_dss_ (L/kg)	1.59 (28.35)	1.18 (27.20)	4.67 (99.48)
	ClCr (mL/min)	112.2 (21.00)	96.6 (18.39)	102.3 (31.16)
Unbound HSK3486	C_max,u_ (ng/mL)	67.1 (15.84)	77.5 (21.81)	74.4 (18.09)
	AUC_0-t,u_ (ng*h/mL)	5.57 (11.05)	6.35 (17.56)	7.83 (34.74)
	AUC_0-inf,u_ (ng*h/mL)	5.95 (10.56)	6.68 (16.85)	9.08 (42.43)
	Clu (L/h)	6754.0 (14.54)	6243.8 (7.44)	4950.1 (38.2)
	Wn_CLu ((L/h)/kg)	101.8 (10.59)	92.4 (18.53)	75.8 (36.97)
	V_d,u_ (L)	40840.1 (42.13)	32018.9 (32.72)	69197.5 (68.48)
	wn_V_d,u_ (L/kg)	615.9 (40.26)	471.2 (32.29)	1059.5 (70.51)
	V_dss,u_ (L)	11303.4 (36.59)	8424.8 (27.44)	27834.6 (93.69)
	wn_V_dss,u_ (L/kg)	169.9 (33.37)	124.6 (29.07)	423.3 (96.09)
	fu	0.96 (0.155)	0.96 (0.189)	1.05 (0.2)
M4	C_max_ (ng/mL)	930.0 (22.08)	1231.9 (34.44)	1003.8 (34.44)
	T_max_ (h)	0.55 (0.52, 0.77)	0.55 (0.53, 0.65)	0.62 (0.53, 2.52)
	t_1/2_ (h)	12.3 (22.96)	14.6 (32.1)	19.3 (39.47)
	AUC_0-t_ (ng*h/mL)	4666.9 (16.32)	6616.4 (41.81)	7496.2 (33.56)
	AUC_0-inf_ (ng*h/mL)	4907.1 (16.11)	7022.0 (42.04)	8571.3 (34.01)
	Ae_0-t_%(%)	29.9 (15.24)	31.2 (20.72)	30.0 (20.61)
	CL_R_0-t_ (L/h)	4.81 (15.69)	3.95 (23.95)	3.04 (14.92)
	wn_CL_R_0-t_ ((L/h)/kg)	0.073 (12.80)	0.058 (30.83)	0.047 (19.72)

Data are means (CV%) for all except T_max_, which is median (range) and fu, which is mean (SD).

C_max_: maximum observed concentration; AUC_0-t_: area under the curve from zero to last time of quantifiable concentration; AUC_0-inf_**_:_** area under the curve from the zero to infinity time; t_1/2_: terminal elimination half-life; T_max_: time to maximum concentration; CL: total clearance; V_d_:distribution volume; V_dss_: the steady state distribution volume; MRT: mean residence time; wn_CL: total clearance adjusted by weight; wn_V_d_: distribution volume adjusted by weight; wn_V_dss_: steady state distribution volume adjusted by weight; fu, free fraction; Ae_0-t_%: accumulative urine excretion rate; CL_R_0-t_: renal clearance; wn_CL_R_0-t_: renal clearance adjusted by weight; ClCr, creatinine clearance rate.

ClCr is based on the Cockcroft–Gault calculation at screening: male ClCr = [140 − age (y)] × weight (kg)/[0.818 × SCr (μmol/L)]; female ClCr= 0.85 × male ClCr.

After the IV bolus dose, the peak plasma concentration of HSK3486 was rapidly achieved, then reached steady state after maintain dose infusion and followed by rapid elimination after infusion was stopped. C_max_ of HSK3486 was similar across three treatment groups, and the AUC increased gradually with the decrease in hepatic function. t_1/2_ was longer in subjects with moderate hepatic impairment (13.2 h) compared to subjects with normal hepatic function (4.17 h) or mild hepatic impairment (3.57 h); however, variability between individuals was large in subjects with moderate hepatic impairment.

GLSM ratios for C_max_ and AUC are summarized in Table 4. C_max_ was 13.6% and 0.4% higher in subjects with mild or moderate hepatic impairment, respectively, compared to normal control group. AUC_0-t_ and AUC_0-inf_ were 12.0% and 10.3% higher in subjects with mild hepatic impairment compared to subjects with normal hepatic function, and 22.3% and 29.5% higher in subjects with moderate hepatic impairment compared to normal control group, respectively.

**Table 4.** Statistical analysis of pharmacokinetic parameters of total and unbound HSK3486 in subjects with varying degrees of hepatic impairment.

	Parameters	Groups	n	Total HSK3486			Unbound HSK3486		
				GLSM	Ratio (%)	90% CI of Ratio (%)	GLSM	Ratio (%)	90% CI of Ratio (%)
Mild vs. Normal	C_max_ (ng/mL)	Mild hepatic impairment	8	7962.3	113.6	(95.2, 135.4)	76.0	114.5	(97.9,133.9)
		Normal hepatic function	8	7012.2			66.4		
	AUC_0-t_ (ng*h/mL)	Mild hepatic impairment	8	655.9	112.0	(95.1, 132.1)	6.26	113.0	(93.1,137.0)
		Normal hepatic function	8	585.5			5.54		
	AUC_0-inf_ (ng*h/mL)	Mild hepatic impairment	8	690.3	110.3	(91.2, 133.3)	6.59	111.2	(89.1,138.8)
		Normal hepatic function	8	626.0			5.93		
									
Moderate vs. Normal	C_max_ (ng/mL)	Moderate hepatic impairment	8	7041.8	100.4	(84.2, 119.7)	73.4	110.6	(94.6,129.3)
		Normal hepatic function	8	7012.2			66.4		
	AUC_0-t_ (ng*h/mL)	Moderate hepatic impairment	8	716.2	122.3	(103.8, 144.2)	7.47	134.7	(111.1,163.4)
		Normal hepatic function	8	585.5			5.54		
	AUC_0-inf_ (ng*h/mL)	Moderate hepatic impairment	8	810.7	129.5	(107.1, 156.6)	8.45	142.6	(114.3,178.1)
		Normal hepatic function	8	626.0			5.93		

GLSM: geometric least squares mean; 90% CI: 90% confidence interval.

Mean unbound (free) drug fraction (fu) was 0.96% in subjects with mild hepatic impairment and 1.05% in subjects with moderate hepatic impairment, which were similar to subjects with normal hepatic function (0.96%). Plasma PK parameters of unbound HSK3486 are summarized in Table 3. The unbound HSK3486 PK parameters change trends with the decrease in hepatic function were similar with that of total HSK3486. C_max_ was 14.5% and 10.6% higher in subjects with mild or moderate hepatic impairment, respectively, compared to normal control group. AUC_0-t,u_ and AUC_0-inf,u_ were 11.2% and 13.0% higher in subjects with mild hepatic impairment compared to normal control group, and 34.7% and 42.6% higher in subjects with moderate hepatic impairment compared to normal control group, respectively.

C_max_ of M4 was similar across three treatment groups, and the AUC increased gradually with the decrease in hepatic function. Mean AUC_0-t_ and AUC_0-inf_ were 41.8% and 43.1% higher in subjects with mild hepatic impairment compared to normal control group, and 60.3% and 74.7% higher in subjects with moderate hepatic impairment compared to normal control group, respectively. t_1/2_ was longer in subjects with moderate hepatic impairment (19.3 h) compared to subjects with normal hepatic function (12.3 h) or mild hepatic impairment (14.6 h).

Cumulative urinary excretion rate (Ae_0-t_%) of M4 was similar across subjects with normal hepatic function (29.9%), mild hepatic impairment (31.2%), or moderate hepatic impairment (30.0%). Renal clearance (CL_R_0-t_ and wn_CL_R_0-t_) showed a decreasing trend with the decrease in hepatic function.

### 3.4. Clinical Effects and pharmacodynamics

**Figure 4.**
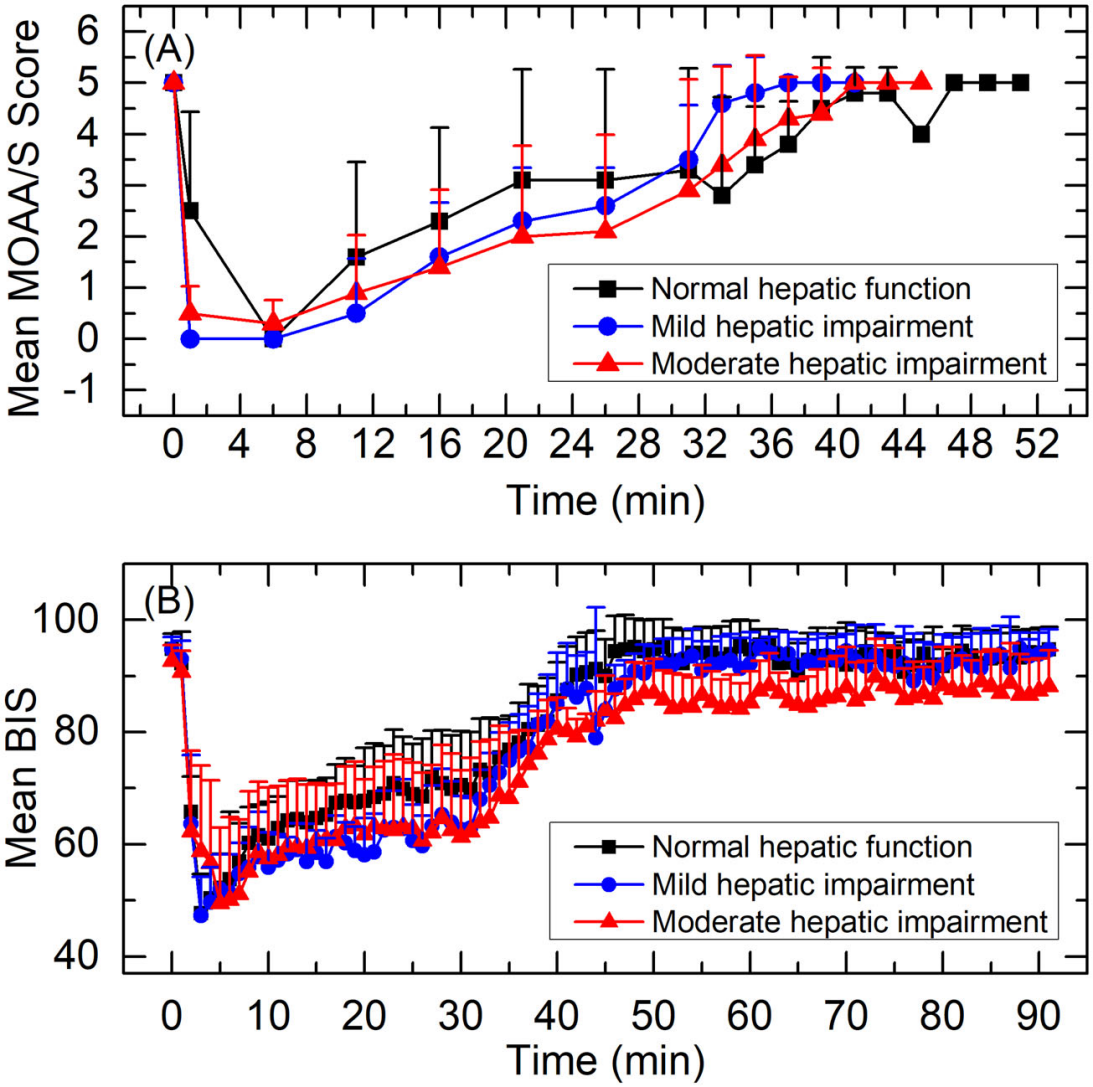
Mean MOAA/S score-time curves (A) and BIS curves (B) for subjects with normal hepatic function, mild hepatic impairment, and moderate hepatic impairment. Data are presented as mean＋standard deviation.

Observed MOAA/S score-time curves are shown in Figure 4(A). All subjects had a MOAA/S score of 5 at baseline (pre-dose). Median time until fully alert, defined as the median time from termination of infusion to the first of three consecutive MOAA/S scores of 5, were 5.02, 2.20 and 4.11 min for subjects with normal hepatic function, mild hepatic impairment or moderate hepatic impairment, respectively.

BIS curves are shown in Figure 4(B). BIS-related parameters are summarised in Table 5. Mean BIS_peak_ was 42.9, 43.5 and 44.9; median T_BISpeak_ was 3.0, 3.5 and 6.0 min; and mean BIS AUC_0-t_ was 7479.1, 7210.7, and 6915.3 for subjects with normal hepatic function, mild hepatic impairment or moderate hepatic impairment, respectively. GLSM ratios for BIS-related parameters are summarised in Table 6. BIS_peak_ and BIS AUC_0-t_ were similar across three treatment groups. 90% CI of GLSM ratios were almost all within 80-125%. T_BISpeak_ in subjects with moderate hepatic impairment was slightly delayed compared to subjects with normal hepatic function or mild hepatic impairment (*p* < 0.05).

**Table 5.** Summary of pharmacodynamic parameters.

BIS parameters		Normal hepatic function (*N* = 8)	Mild hepatic impairment (*N* = 8)	Moderate hepatic impairment (*N* = 8)
BIS_peak_	Mean(SD)	42.9(7.14)	43.5 (10.04)	44.9(13.63)
	Median	44	43	41.5
	Min, Max	28.0,51.0	23.0,58.0	34.0,74.0
T_BISpeak_(min)	Mean(SD)	3.75(1.17)	8.73(14.29)	7.48(5.27)
	Median	3	3.5	6
	Min, Max	2.97,6.00	2.80,44.00	3.00,20.00
BIS AUC_0-t_	Mean(SD)	7479.1 (343.0)	7210.7 (255.5)	6915.3 (438.7)
	Median	7586.3	7242.9	6892.0
	Min, Max	6853.8,7850.4	6857.3,7537.6	6391.8,7710.6
Time until fully alert (min)	Mean (SD)	5.27(5.77)	2.62(1.74)	4.85(4.07)
	Median	5.02	2.2	4.11
	Min, Max	0.00,16.1	0.00,6.00	0.05,10.1

BIS_peak_: BIS peak value (the lowest BIS value); T_BISpeak_: time to BIS peak; BIS AUC_0-t_: area under the BIS curve from zero to last collection time.

**Table 6.** Statistical analysis of pharmacodynamic parameters in subjects with varying degrees of hepatic impairment.

	Parameters	Groups	n	GLSM	Ratio (%)	90% CI of Ratio (%)
Mild vs. Normal	BIS_peak_	Mild hepatic impairment	8	42.3	100	(80.9, 123.7)
		Normal hepatic function	8	42.3		
	BIS AUC_0-t_	Mild hepatic impairment	8	7206.8	96.5	(92.5, 100.6)
		Normal hepatic function	8	7472.1		
Moderate vs. Normal	BIS_peak_	Moderate hepatic impairment	8	43.3	102.5	(82.9, 126.8)
		Normal hepatic function	8	42.3		
	BIS AUC_0-t_	Moderate hepatic impairment	8	6903.4	92.4	(88.6, 96.4)
		Normal hepatic function	8	7472.1		
	Parameters	Groups	n	Median	90% CI of the Difference	*P*-Value
Mild vs. Normal	T_BISpeak_	Mild hepatic impairment	8	3.5		
		Normal hepatic function	8	3	(−1.00,1.03)	.738
Moderate vs. Normal	T_BISpeak_	Moderate hepatic impairment	8	6		
		Normal hepatic function	8	3	(1.00,3.85)	.015

BIS_peak_: BIS peak value (the lowest BIS value); T_BISpeak_: time to BIS peak; BIS AUC_0-t_: area under the BIS curve from zero to last collection time; GLSM: geometric least squares mean; 90% CI: 90% confidence interval.

### 3.5. Pk-PD relationship

**Figure 5.**
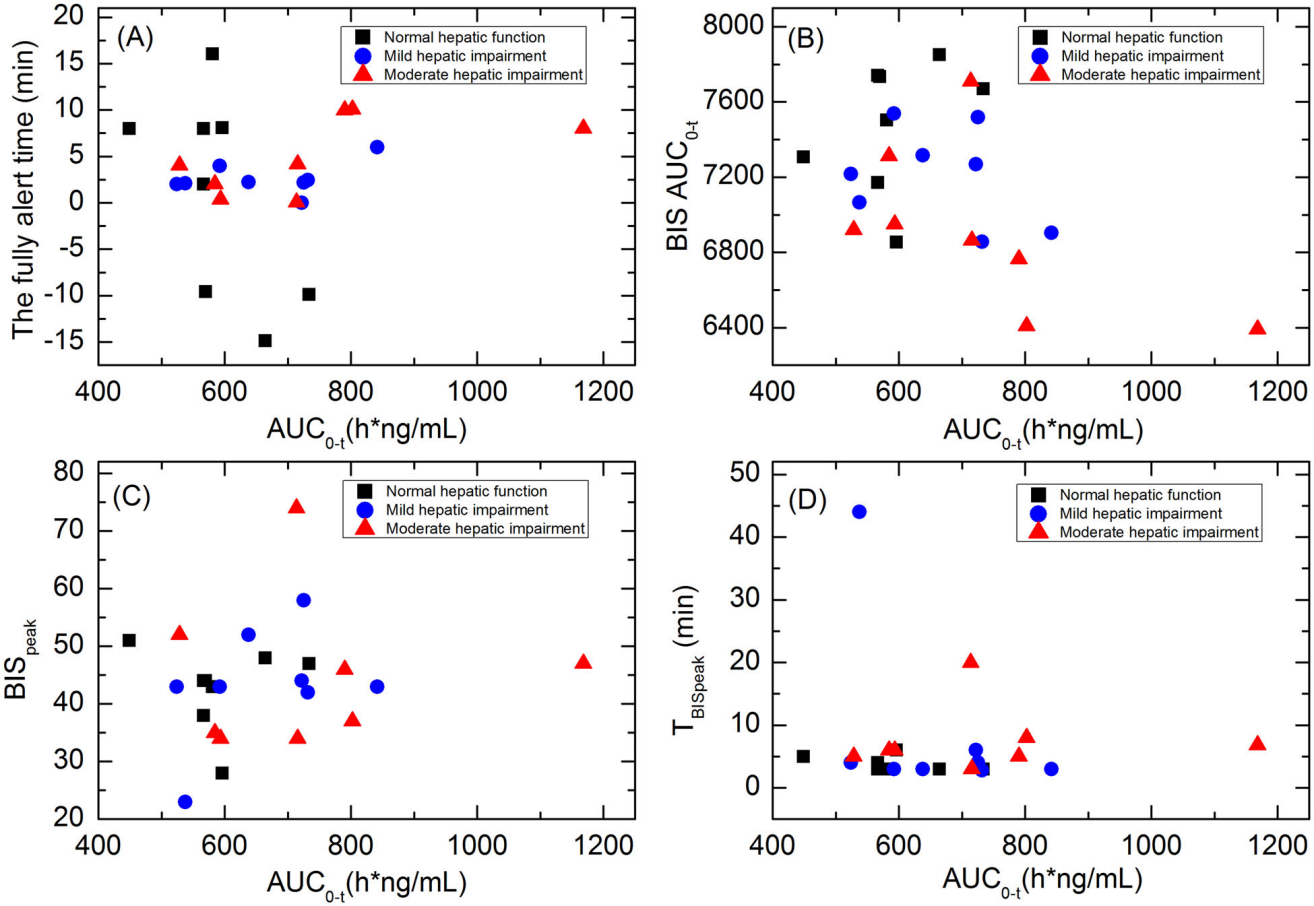
Scatterplots showing the correlations between HSK3486 exposure (AUC_0-t_) and time until fully alert (A); BISAUC_0-t_ (B); BIS_peak_ (C); and T_BISpeak_ (D) in subjects with normal hepatic function, mild hepatic impairment, and moderate hepatic impairment.

Scatterplots showing the correlations between HSK3486 exposure (AUC_0-t_) and time until fully alert, BISAUC_0-t_, BIS_peak_ and T_BISpeak_ for subjects with normal hepatic function, mild hepatic impairment, or moderate hepatic impairment are shown in Figure 5. There were no significant correlations between HSK3486 exposure and these PD parameters.

**Figure 6.**
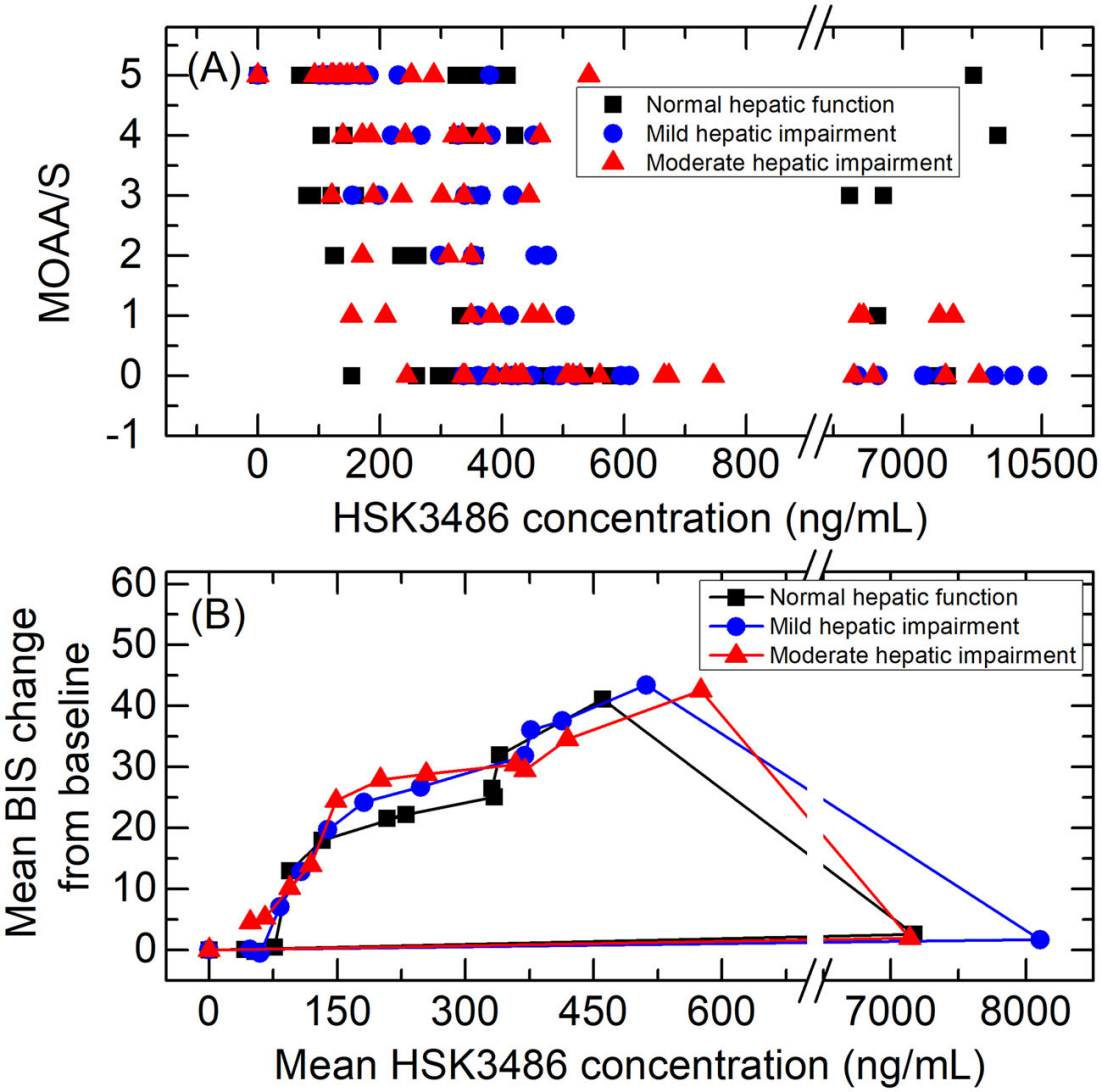
Scatterplots showing the correlations between HSK3486 plasma concentration and MOAA/S scores (A), and mean HSK3486 plasma concentrations and mean BIS change from baseline (B) in subjects with normal hepatic function, mild hepatic impairment, and moderate hepatic impairment.

Scatterplots showing the correlations between HSK3486 plasma concentration and MOAA/S scores are shown in Figure 6(A). When MOAA/S scores were ≤1, HSK3486 concentrations (median (min, max)) in subjects with normal hepatic function, mild hepatic impairment or moderate hepatic impairment were 423.5 (154, 8120), 503 (337, 10400) and 512 (153, 8920) ng/ml, respectively. After infusion, when MOAA/S scores were 5, HSK3486 concentrations (median (min, max)) were 158 (68.7, 8790), 143 (101, 230), and 136 (92.6, 251) ng/ml, respectively, and there were observable trends.

Scatterplots showing the correlations between mean HSK3486 plasma concentration and mean BIS change from baseline in subjects with normal hepatic function, mild hepatic impairment, or moderate hepatic impairment are shown in Figure 6(B). Findings were similar across the three cohorts.

## 4. Discussion

This study explored the impact of hepatic impairment on HSK3486 safety, PK and PD in Chinese subjects. The results indicated that HSK3486 was safe and well tolerated in subjects with normal hepatic function, mild hepatic impairment or moderate hepatic impairment, and the AUC of HSK3486 gradually increased with the decrease in hepatic function; however, the degree of hepatic impairment had little effect on HSK3486 PD (MOAA/S, BIS).

There were no serious AEs and no deaths during the study. No AEs led to subject withdrawal from the study. Subjects with moderate hepatic impairment experienced the highest incidence of AEs, likely due to their poorer overall health status. As common adverse reactions of anaesthetic drugs, respiratory depression and hypotension are the only two AEs occurred in all three groups. The incidence of hypotension was the same across subjects with normal hepatic function, mild hepatic impairment, or moderate hepatic impairment (25%), but the incidence of respiratory depression was highest in subjects with moderate hepatic impairment (50% vs.12.5% and 25%). The increased incidence of respiratory depression may be related to degree of hepatic impairment, and subjects with moderate hepatic impairment should be carefully monitored when administered HSK3486 for anaesthesia. Subjects in this study experienced a higher incidence of AEs than subjects in previous studies of HSK3486, likely due to the smaller sample size.

In a previous trial investigating the mass balance of HSK3486 in healthy subjects, mean recovery of radioactivity in urine and faeces was 84.6% and 2.65%, respectively. Total plasma radioactivity concentrations were higher than unchanged HSK3486 plasma concentrations, indicating the presence of circulating metabolites. The major circulating metabolite was identified as a glucuronide conjugate of HSK3486 (M4) (79.3%), while only 3.97% of the total radiation exposure was due to unchanged HSK3486. Thus, similar to propofol, extensive metabolic clearance to inactive metabolites by UDP-glucuronosyltransferases (UGTs) and CYP enzymes in the liver was considered the major contributor to total body clearance of HSK3486. *In vitro* studies imply that CYP2B6 is the major CYP enzyme that mediates HSK3486 metabolism [2, 12].

Hepatic impairment can influence the function of many CYP enzymes, including CYP2B6, while UGT activity may be affected to a lesser extent [14]. Therefore, changes in CYP2B6 activity were likely responsible for the slight increase in HSK3486 exposure (<30% for AUC and <15% for C_max_) in subjects with mild and moderate hepatic impairment. These increases in HSK3486 exposure were not considered clinically relevant and do not warrant dose adjustments.

Similar to propofol, HSK3486 binds extensively to plasma proteins. *In vivo*, approximately 99% of HSK3486 is bound to albumin, which is the major binding component in human plasma [18]. Liver dysfunction is associated with reduced serum albumin levels, implying that concentrations and pharmacological effects of unbound HSK3486 may be elevated in subjects with mild or moderate hepatic impairment [19]. In the present study, mean albumin concentrations were similar in subjects with normal hepatic function (39.71 g/L) and mild hepatic impairment (42.30 g/L) but higher than in subjects with moderate hepatic impairment (33.61 g/L). However, the unbound fraction of HSK3486 was similar across the three cohorts, suggesting that the slightly lower albumin concentrations in subjects with moderate hepatic impairment had little relevant effect on HSK3486 clearance.

Drugs that act on the central nervous system can lead to episodes of hepatic encephalopathy [20–22]. Previous studies have investigated the impact of hepatic function on propofol PK. Findings revealed no safety concerns associated with propofol sedation in patients with cirrhosis, there was no evidence of newly developed hepatic encephalopathy, and propofol PK was not significantly different between control subjects and those with cirrhosis. These data suggest that propofol can be used without major dose reductions in cirrhotic patients [21, 23–26]. Accordingly, the present study showed the AUC of HSK3486 increased gradually with the decrease in hepatic function, however, degree of hepatic impairment had little effect on HSK3486 PD (MOAA/S, BIS), and no hepatic encephalopathy was observed.

This study was associated with several limitations. First, subjects with severe hepatic impairment (Child-Pugh C) were not included, and the findings from this study should not be extrapolated. Second, the sample size was small. Future studies with a larger number of cases are required to support our results.

## 5. Conclusion

HSK3486 at an IV bolus dose of 0.4 mg/kg and a maintenance infusion of 0.4 mg/kg/h was safe and well tolerated by all subjects. There were no clinically relevant increases in HSK3486 exposure (C_max_ and AUC) or clinical effects changes in subjects with mild or moderate hepatic impairment compared to normal control. These data imply no HSK3486 dose adjustment is required in subjects with mild or moderate hepatic impairment.

## Author contributions

Study design: YH, XJL, HC, YHD, CLX

Data collection: YH, XJL, JRL, CLX, HC, WBZ, HZ, MW, CYL, XXZ, JFL, YHD

Data analysis: PKY, NW, XL, SPM, XW

Drafting of article: YH, XJL, YHD

Revision of article: PKY, NW, XL, SPM, XW, YHD

Approval of the final version of the article and agreement to accountability: all authors

## Data Availability

The data that support the findings of this study are available on request from the corresponding author Xiaojiao Li.
